# A High Load of Non-neutral Amino-Acid Polymorphisms Explains High Protein Diversity Despite Moderate Effective Population Size in a Marine Bivalve With Sweepstakes Reproduction

**DOI:** 10.1534/g3.112.005181

**Published:** 2013-02-01

**Authors:** Estelle Harrang, Sylvie Lapègue, Benjamin Morga, Nicolas Bierne

**Affiliations:** * Ifremer, Laboratoire de génétique et pathologie, 17390 La Tremblade, France; † Université Montpellier 2, 34095 Montpellier cedex 5, France; ‡ CNRS - Institut des Sciences de l'Evolution, UMR5554, Station Méditerranéenne de l’Environnement Littoral, 34200 Sète, France

**Keywords:** nucleotide polymorphism, marine bivalve, deleterious mutations, genetic load, *Ostrea edulis*

## Abstract

Marine bivalves show among the greatest allozyme diversity ever reported in Eukaryotes, putting them historically at the heart of the neutralist−selectionist controversy on the maintenance of genetic variation. Although it is now acknowledged that this high diversity is most probably a simple consequence of a large population size, convincing support for this explanation would require a rigorous assessment of the silent nucleotide diversity in natural populations of marine bivalves, which has not yet been done. This study investigated DNA sequence polymorphism in a set of 37 nuclear loci in wild samples of the flat oyster *Ostrea edulis*. Silent diversity was found to be only moderate (0.7%), and there was no departure from demographic equilibrium under the Wright-Fisher model, suggesting that the effective population size might not be as large as might have been expected. In accordance with allozyme heterozygosity, nonsynonymous diversity was comparatively very high (0.3%), so that the nonsynonymous to silent diversity ratio reached a value rarely observed in any other organism. We estimated that one-quarter of amino acid-changing mutations behave as neutral in *O. edulis*, and as many as one-third are sufficiently weakly selected to segregate at low frequency in the polymorphism. Finally, we inferred that one oyster is expected to carry more than 4800 non-neutral alleles (or 4.2 cM^−1^). We conclude that a high load of segregating non-neutral amino-acid polymorphisms contributes to high protein diversity in *O. edulis*. The high fecundity of marine bivalves together with an unpredictable and highly variable success of reproduction and recruitment (sweepstakes reproduction) might produce a greater decoupling between *Ne* and *N* than in other organisms with lower fecundities, and we suggest this could explain why a higher segregating load could be maintained for a given silent mutation effective size.

Marine bivalves are recognized as being among the most polymorphic of all animal species. First observed with allozyme markers (Ward *et al.* 2000; Bazin *et al.* 2006), this high polymorphism now appears to be confirmed at the nucleotide level (Sauvage *et al.* 2007; Arias *et al.* 2009; Zhang and Guo 2010). However, the cause of the extreme heterozygosity of marine bivalves has remained controversial, and its investigation has been hampered by several factors.

First, the diversity of functional markers (*e.g.*, allozymes or nonsynonymous polymorphisms) depends on the concomitant effects of population size and selective constraints on these markers, whereas the diversity of nonfunctional markers (*e.g.*, synonymous or noncoding polymorphisms) only depends on the population size (Kimura 1983). Ideally, genetic diversity should only be estimated using nonfunctional polymorphisms (*i.e.*, silent diversity).

Second, although silent diversity has now been evaluated in a few bivalve species, these studies were designed to discover single-nucleotide polymorphisms (SNPs) and used individuals sampled from hatcheries rather than the wild, precluding an inference of the frequency of mutations in natural populations. Furthermore, the use of SNP density as a measure of diversity does not provide an accurate statistic for rigorous comparison among species and can be misleading.

Third, extreme polymorphism is sometimes prone to selectionist interpretations. This was true at the allozyme time, during which numerous studies questioned neutrality of these markers in marine mollusks (Koehn and Shumway 1982; Koehn 1990; Karl and Avise 1992; Mitton 1993, 1997; Riginos *et al.* 2002). More recently this has been reiterated in some surveys of DNA sequence polymorphism of protein-coding genes (Moy *et al.* 2008; Wharam *et al.* 2008; Costa *et al.* 2009; Parisi *et al.* 2009). It is therefore crucial to build a clearer picture by evaluating the genomic average of diversity of various categories of substitutions from a large panel of genes in different species of bivalves. Only knowledge of this kind would allow us to confidently say that the diversity of a specific protein was extreme.

Finally, and perhaps most importantly, large variation in reproductive success owing to a skewed offspring distribution can lead to effective population sizes several orders of magnitude below census numbers and the effective size of marine populations might not be as large as was first anticipated (Hedgecock 1994; Hedgecock and Pudovkin 2011). A skewed offspring distribution can also complicate the interpretation of descriptive statistics of genetic variation as this creates deviation from the standard Wright-Fisher model and the Kingman’s coalescent (Sargsyan and Wakeley 2008, Eldon and Wakeley 2009, Der *et al.* 2012). All these caveats may have fueled the doubt cast on the simplest hypothesis: that the extreme protein diversity of marine mollusks is a direct consequence of their large population size. The present paper addresses this doubt.

We studied DNA sequence polymorphism in the flat oyster *Ostrea edulis* by direct resequencing of 37 loci identified from expressed sequence tag (EST) libraries (Morga *et al.* 2011, 2012). Samples were taken from different natural populations throughout the distribution area of *O. edulis* in Europe. The aim of this study was first to obtain a new estimate of silent diversity based on wild samples in an additional species of bivalve and second to verify whether purifying selection could be relaxed in marine bivalves, as suggested by Sauvage *et al.* (2007), by further investigating the potentially non-neutral nature of segregating nonsynonymous polymorphisms.

## Materials and Methods

### Sampling and Molecular Protocols

We used 16 oysters from four different natural populations collected on the Atlantic and Mediterranean coasts, in Italy, Greece and France. Genomic DNA was extracted from samples of gill tissue using the Wizard DNA Clean-Up System (Promega) according to the manufacturer’s recommendations. Quality and concentration were assessed on a 1% agarose gel and by using an Eppendorf BioPhotometer. Concentrations were equilibrated to 100 ng.µL^−1^ per DNA sample.

Primers were designed from 114 EST sequences by using the online software Primer3 (Rozen and Skaletsky 2000). Expected fragment lengths in the absence of introns varied between 160 bp and 660 bp depending on the EST used. The polymerase chain reaction (PCR) protocol was the same as in Sauvage *et al.* (2007). Purified PCR products were then sequenced with both the forward and the reverse primers, using the ABI Prism BigDye v3 Terminator Cycle sequencing Kit (Applied Biosystems) in an ABI 3130 genetic analyzer (Applied Biosystems). A second PCR amplification and a second sequencing reaction allowed the removal of most of the artifactual mutations inherently produced during the experiments.

### Analysis of DNA sequence polymorphism

Sequence alignment was performed with ClustalW via the BioEdit interface (Hall 1999). Verification by eye and multiple alignments of sequences obtained from different PCR and sequencing reactions for the same individual were used to correct for artifactual mutations. All sites with two simultaneous fluorescent signals were carefully individually inspected and considered to be heterozygous sites if the intensity of the least intense peak was greater than 30% of the intensity of the most intense peak. The cDNA was used to identify intronic regions. A BLAST homology search was performed on GenBank (http://blast.ncbi.nlm.nih.gov/Blast.cgi) to identify orthologous sequences which, together with ORF-Finder (Rombel *et al.* 2002), enabled the annotation of open reading frames (ORFs) and untranslated regions (UTRs). Annotations were further investigated and confirmed with Artemis (Rutherford *et al.* 2009). As we used direct sequencing, sequences stopped at the first heterozygous indel, sometimes producing sequences of variable lengths (L). For this reason, some sequences were too short for the analysis, and we obtained variable sample sizes. The validity of each SNP was checked individually on nucleotide sequences and sequence alignments.

SNPs were classified as synonymous and nonsynonymous in coding regions and as noncoding in intronic and UTR regions. Synonymous and noncoding SNPs were classified as silent. Genetic diversity was analyzed with DnaSP v5.10 (Librado and Rozas 2009) and double checked with homemade programs of the Montpellier Bioinformatics Biodiversity platform. We computed the nucleotide diversity (*π*, Nei 1987) and Watterson’s theta (*θ*, Watterson 1975) for each site category: synonymous (*π_s_*, *θ_s_*), nonsynonymous (*π_n_*, *θ_n_*), noncoding (*π_nc_*, *θ_nc_*), and silent (*π_si_*, *θ_si_*). Departure from the expectations of mutation/drift equilibrium under the Wright-Fisher model was evaluated with silent sites, using Tajima’s D statistic (Tajima 1989), which is a standardized measure of the difference between the two diversity estimators (*π* and *θ*). Genetic structure, as measured by F_ST_ statistics (Wright 1951), was analyzed with Arlequin v3.5 (Excoffier *et al.* 2005) among all populations, and between Mediterranean and Atlantic populations. ORFs were used to compute the GC content at the third codon position (GC3) and to measure the effective number of codons (ENC) with DnaSP v5.10 (Librado and Rozas 2009).

### Estimating the fractions of nonsynonymous mutations that are neutral, weakly selected, or strongly deleterious

We used the method of Fay *et al.* (2001) to partition amino acid mutations into three categories: neutral, slightly selected, and strongly deleterious. The format of our dataset did not allow us to estimate a more detailed distribution of fitness effects (*e.g.*, Eyre-Walker *et al.* 2006). Although simpler, the method of Fay *et al.* (2001) does not rely on a specific population genetic model (*e.g.*, the Wright-Fisher model), and in the spirit of the McDonald-Kreitman approach (1991), it capitalizes on the fact that neutral and potentially selected sites are interspersed with each other throughout a gene. Such sites are therefore expected to have the same evolutionary history and sampling (*i.e.*, a shared genealogy). A possible deviation from the standard coalescent, as could be expected in marine species with a skewed offspring distribution (Eldon and Wakeley 2009), is not expected to bias the method, which simply assumes shared genealogies for the two categories of mutations whatever their shapes (*e.g.*, standard, multifurcating, star-like...). The neutral class, *f_0_*, was estimated from common SNPs; the slightly selected class, *f_1_*, accounted for the excess of low frequency amino acid SNPs; and the strongly deleterious class, *f_2_*, was deduced from the synonymous diversity as those nonsynonymous SNPs that were lacking from the sample. SNPs were classified into frequency categories: those with a minor variant frequency less than 0.1, those with a frequency between 0.1 and 0.2, and those with a frequency greater than 0.2. In addition, following the idea of Fay *et al.* (2001) that deleterious mutations are not expected to spread on a wide geographical scale, we also classified SNPs as shared or nonshared between populations or between the Atlantic Ocean and the Mediterranean Sea. The comparison of the *θ_n_*/*θ_si_* ratio among categories allowed *f_0_*, *f_1_*, and *f_2_* to be estimated (Fay *et al.* 2001).

The number of nonsynonymous SNPs expected if they were all neutral was estimated from the observed number of silent SNPs and used to calculate the proportion of non-neutral nonsynonymous SNPs in the sample. Estimating the number of heterozygote non-neutral amino-acid mutations an individual carries would require knowledge of the frequency of these mutations in the population. An underestimation and thus conservative estimate can nonetheless be obtained following Fay *et al.* (2001) by assuming non-neutral nonsynonymous alleles are present only once in the sample.

## Results

### DNA polymorphism in *O. edulis*

From the 114 loci that we attempted to amplify, 13 presented no amplification signal, which could be explained by the presence of an intron within the priming sequences or a long intron between the two primers, and 47 presented a multibanded pattern. Among the 54 loci that produced a single-banded pattern on agarose gel, 10 produced unreadable electropherograms (overlapping peaks), two were composed of very large introns (>1000 bp), which precluded a forward/reverse sequencing double check, and two could not be aligned with their corresponding cDNA, indicating a lack of specificity of the primers or PCR conditions. Forty loci produced usable sequence sets and were long enough to be annotated and deposed in the GenBank database (accession nos. JN680816 to JN680855). Among these, 23 were composed of one to three large introns, with an individual length from 92 to 983 bp and a total length per locus from 92 to more than 1250 bp. The primers are listed in Supporting Information, Table S1 and a polymorphism analysis of the 40 loci is shown in Table S2. Three loci were mitochondrial (JN680816, JN680817, JN680826) and were discarded from subsequent analyses due to the specific characteristics of mitochondria (uniparental inheritance, nonrecombining, greater selective constraint on mitochondrial than nuclear proteins), as is usual practice in similar studies, including those with which we compare our data in the *Discussion*. A total of 16,525 base pairs of the European flat oyster genome were successfully sequenced and analyzed (37 nuclear loci, Table S2). Twenty-six of the 37 loci contained at least one noncoding region (intron and/or UTR), and one locus was entirely noncoding. A total of 283 SNPs were identified (Table S3) of which 28% were nonsynonymous, 12% were synonymous, and 60% were noncoding. Only one locus was monomorphic. An average density of 1 SNP every 76 bp was observed in coding regions and 1 every 47 bp in noncoding regions, which are comparable densities to those observed in the cupped oyster *Crassostrea gigas* (Sauvage *et al.* 2007).

The average nucleotide diversity at synonymous sites was *π_s_* = 0.006 (range, 0−0.08) and the average Watterson’s theta of *θ_s_* = 0.005. The average nucleotide diversity at non-coding sites was *π_nc_* = 0.005 (range, 0−0.017) with an average Watterson’s theta of *θ_nc_* = 0.006. Synonymous and noncoding diversities were not significantly different from each other (nucleotide diversity *t* = 0.97; *df* = 36; *P* = 0.34; Watterson’s theta: *t* = 0.29; *df* = 36; *P* = 0.77), so we combined the two categories of polymorphism into a single category (silent mutations). The average nucleotide diversity at silent sites was *π_si_* = 0.0067 (range, 0−0.08) with an average Watterson’s theta of *θ_si_* = 0.0065. The similarity between the two estimators (*π_si_* and *θ_si_*) suggests an absence of any strong deviation from the expectation of demographic equilibrium under a Wright-Fisher model.

**Figure 1:**
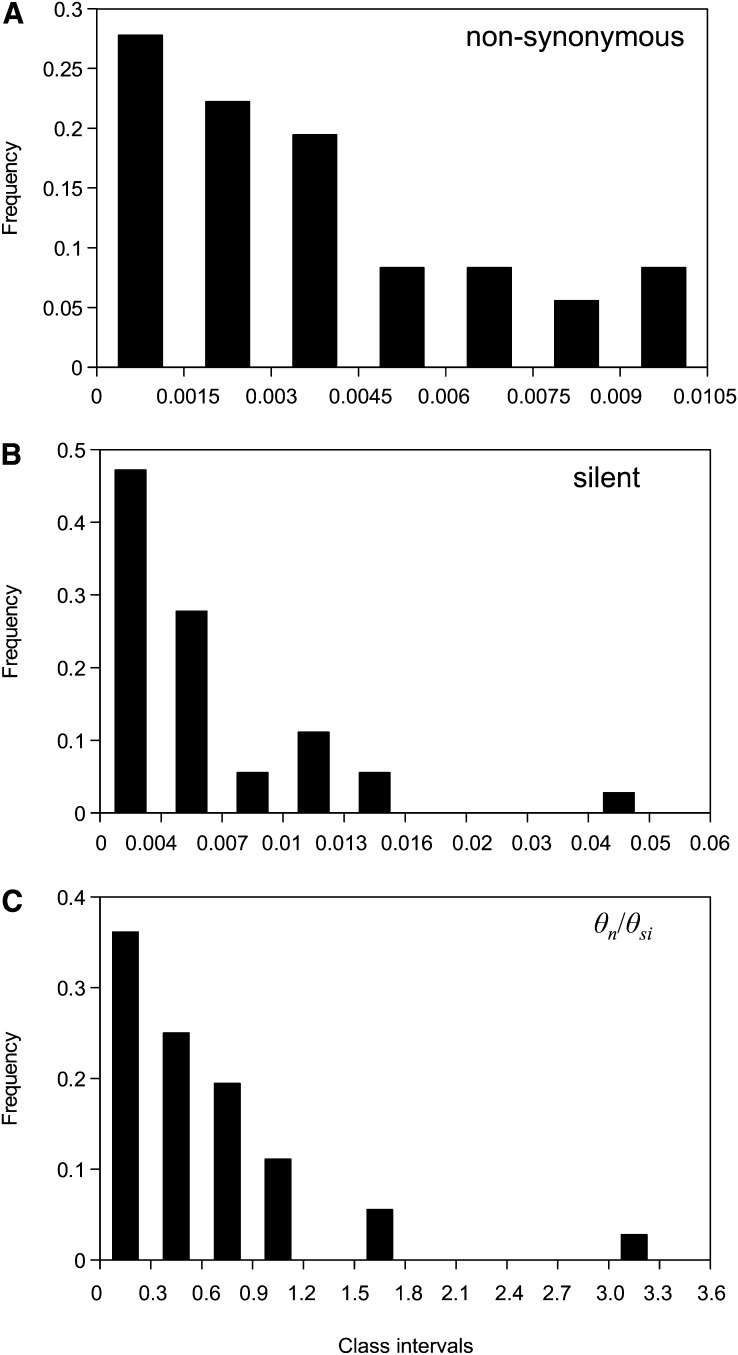
Distribution of Watterson’s theta (A) at nonsynonymous sites (*θn*) and (B) at silent sites (*θsi*) and (C) distribution of the nonsynonymous-to-silent diversity ratio (*θn*/*θsi*) across the 37 loci.

The average nucleotide diversity at nonsynonymous sites was *π_n_* = 0.0025 (range, 0−0.014) and was lower than the Watterson’s theta, which was *θ_n_* = 0.0035. This illustrates that nonsynonymous polymorphisms tend to segregate at low frequency. The nonsynonymous to silent diversity ratios were *π_n_*/*π_si_* = 0.38 and *θ_n_*/*θ_si_* = 0.56. To be conservative in our conclusions, and to display a genome-wide trend without the influence of specific loci that might cause downward *θ_si_* or upward *θ_n_*/*θ_si_* bias, we plotted the distributions of *θ_n_*, *θ_si_*, and *θ_n_*/*θ_si_* across loci (Figure 1). Two loci with a high *θ_n_* (JN680851 and JN680855) were removed from further analysis as they could have artificially increased our estimates of the genetic load, although the diversity estimates were virtually unchanged (*π_n_* = 0.0025; *π_si_* = 0.0071; *π_n_*/*π_si_* = 0.36; *θ_n_* = 0.0034; *θ_si_* = 0.0066; *θ_n_*/*θ_si_* = 0.52). One locus showed a surprisingly high silent diversity. This locus was also the only one exhibiting a significant positive Tajima’s D, which would suggest it could be under balancing selection or that we might have amplified paralogous sequences. Including this locus in the analysis makes our conclusion of a low silent diversity conservative.

Figure 1 suggests that if we have a biased representation of the genetic diversity of the flat oyster genome, it would be caused by global bias in the choice of the loci analyzed. To check for such a bias, we verified whether the results were affected by the functional categories of the gene surveyed and by codon usage, which can be used as a proxy for expression levels as well as selective constraints on the protein (Stoletzki *et al.* 2005; Bierne and Eyre-Walker 2006; Sauvage *et al.* 2007). Genes were clustered into eight categories according to their putative biological function predicted by NCBI according to Gene Ontology (Figure S1). The distribution of *θ_n_*/*θ_si_* across the eight functional categories is presented in supplementary Figure S2. A similarly high *θ_n_*/*θ_si_* was estimated for every functional group, including ribosomal proteins, which are known to be highly constrained in other species. Representativeness of the genome diversity was also checked by computing GC3 and ENC on our set of 37 loci and on the full EST database (Figure S3). Codon bias proved to be low (high value of ENC) and GC3 moderately low. The distribution of the two measures was not significantly different between the two datasets. We also tested whether codon biases were correlated with diversity indices. Neither *θ_s_* nor *θ_nc_* or *θ_n_* were significantly correlated with ENC (*θ_s_*: *r* = −0.12, *P* = 0.49, *θ_nc_*: *r* = −0.04, *P* = 0.79, *θ_n_*: *r* = 0.13, *P* = 0.45). Finally, we verified if some errors in the annotation of the ORFs could have biased the results by removing every loci with an annotated UTR, and the results were not profoundly modified (*π_n_* = 0.0023, *θ_n_* = 0.003, *π_n_*/*π_si_* = 0.31, and *θ_n_*/*θ_si_* = 0.48).

Tajima’s D was computed with silent mutations for each polymorphic locus. Negative values were obtained for 24 loci (ranged from −2.01 to −0.15) and positive values for 10 loci (ranged from 0.12 to 2.43). Among these values, none were statistically different from zero after a correction for multiple testing. The average Tajima’s D was D = −0.38 with the 37 loci dataset and D = −0.5 with the 35 loci dataset.

Distribution of F_ST_ values for nonsynonymous and silent SNPs between all populations, and between Mediterranean and Atlantic populations, are presented on Figure S4. Although a large variance is observed among loci due to the small size of our samples, this analysis shows that the genetic differentiation was low overall and did not differ between the two categories of mutations.

### Selective constraint and the load of segregating non-neutral mutations

**Figure 2:**
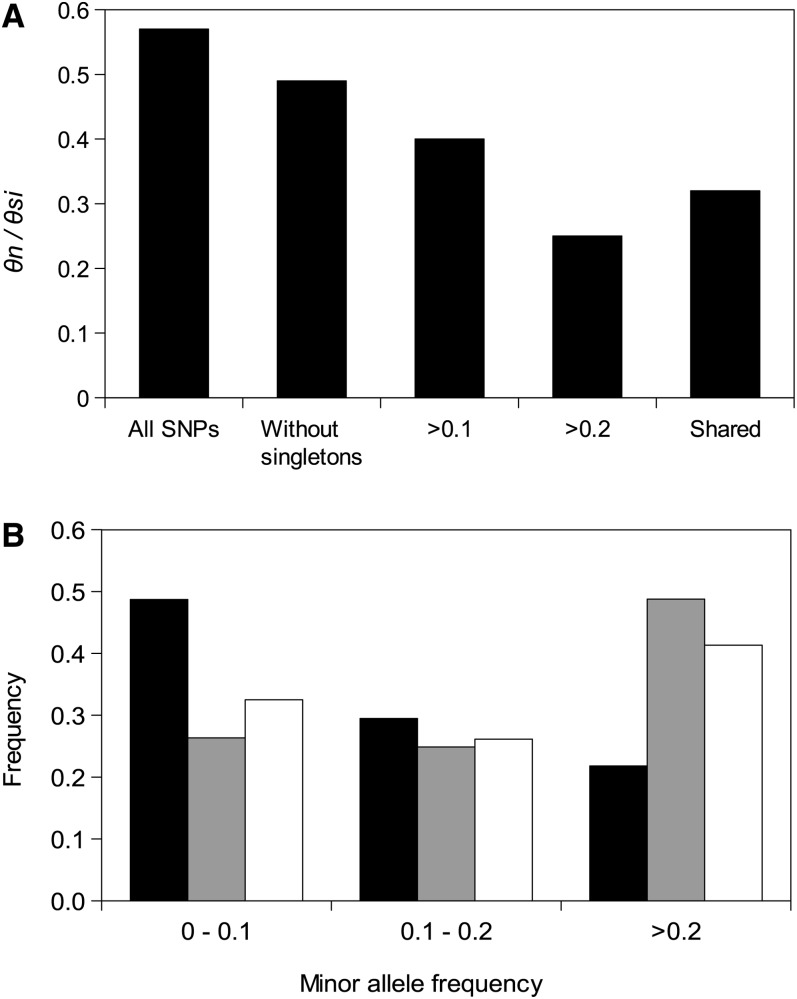
(A) Nonsynonymous-to-silent diversity ratio (*θn*/*θsi*) in the *O. edulis* nuclear genome. All SNPs: full dataset; without singletons: when singletons are removed from the dataset; >0.1: SNPs with a minor variant frequency greater 0.1; >0.2: SNPs with a minor frequency greater than 0.2.; shared: SNPs shared between at least two populations from the Atlantic or the Mediterranean Sea. (B) Allele frequency spectrum of nonsynonymous (dark), silent (gray), and expected under the neutral Wright-Fisher model (light). 0−0.1: SNPs with a minor variant frequency below 0.1; 0.1−0.2: SNPs with a minor variant frequency between 0.1 and 0.2; >0.2: SNPs with a minor variant frequency greater than 0.2. 35 loci dataset.

Figure 2A shows the *θ_n_*/*θ_si_* ratio for various frequency categories of SNPs, and Figure 2B shows the allele frequency spectrum of nonsynonymous and silent mutations, as well as the expectations of the mutation/drift equilibrium under the Wright-Fisher model. This shows that: (1) the frequency spectrum of silent SNPs did not depart from the neutral expectation at mutation/drift equilibrium under the Wright-Fisher model; (2) there is an excess of nonsynonymous mutations segregating at low frequency, suggesting that a large fraction of the amino acid changing SNPs are slightly selected; (3) the *θ_n_*/*θ_si_* ratio decreases when rare variants or when population-specific SNPs are removed, and it converges at a value of 23%, which can be used as an estimate of *f_0_* (Fay *et al.* 2001), the fraction of amino acid-changing mutations that behave as neutral (or at least as silent mutations do). We then estimated that 44% of amino acid mutations are strongly deleterious and not found in the sample, and 33% are slightly selected and segregate at low frequency.

We observed 68 nonsynonymous SNPs in the sample (35 loci dataset) whereas 110 would have been expected from the number of silent SNPs if nonsynonymous mutations were all neutral. From the calculation above, a fraction *f_0_* = 0.23 of nonsynonymous mutations are estimated to be neutral, which allowed us to infer that 25 of the 68 observed nonsynonymous SNPs were expected to be neutral and the remaining 43 were expected to be non-neutral in our sample of 16 oysters. By assuming non-neutral nonsynonymous alleles are present only once in the sample (Fay *et al.* 2001), we could conservatively estimate (*i.e.*, underestimate) that the number of non-neutral nonsynonymous heterozygote mutations carried by an individual in the 35 loci surveyed was 3.2. Assuming there are 10^7^ nonsynonymous sites in the oyster genome, we inferred that one diploid genome can be expected to carry more than ~4800 non-neutral mutations (or 2400 non-neutral mutations per haploid genome). Assuming a genetic map of 575 cM (Lallias *et al.* 2007) we therefore deduced the density of non-neutral mutations in the oyster genome to be 4.2 cM^−1^.

## Discussion

The explanation for the extreme protein heterozygosity of marine bivalves has been hotly debated in the past, at the heart of the neutralist−selectionist controversy, but the question has never been answered definitively. With the switch to DNA based molecular techniques to monitor genetic diversity, the time-lag to collect within-species genomic data in nonmodel organisms has led interest on the issue to dissipate. The simplest explanation—that a high diversity is the result of a large population size—has persisted as the valid interpretation. This was indeed the most logical explanation for the extreme diversity observed in *Ciona* sea squirts (Small *et al.* 2007; Tsagkogeorga *et al.* 2012), another genus of ocean-dwelling broadcast spawners. As a high density of SNPs has been observed in the first marine bivalves studied (Sauvage *et al.* 2007; Li *et al.* 2009; Zhang and Guo 2010; Kim *et al.* 2011), the same explanation was proposed (Sauvage *et al.* 2007), but perhaps too hastily.

### Moderate silent diversity in a marine bivalve

In the present study, the density of SNPs was comparable with those observed in other bivalves (1 SNP every 50 bp). However, we were able to obtain estimates of nucleotide diversity to use in a rigorous comparison with published results on other species. The silent nucleotide diversity estimated in *Ostrea edulis* (*π_si_* = 0.007) was not extreme and appears rather moderate when compared with the champions of DNA polymorphism such as some *Caenorhabditis* nematodes [*C. remanei*: *π_s_* = 0.045 (Cutter *et al.* 2006), *Caenorhabditis sp. 5*: *π_s_* = 0.068 (Wang *et al.* 2010)] or *Ciona* sea squirts [*C. savignyi*: *π_s_* = 0.08 (Small *et al.* 2007) *C. intestinalis sp. B*: *π_s_* = 0.057 (Tsagkogeorga *et al.* 2012)]. The simplest explanation is that the effective population size of *O. edulis* is not as large as might have been expected, which could be due to an intrinsically low effective size that remained roughly constant for a long period, or the consequence of a departure from demographic equilibrium (*i.e.*, a population bottleneck). At first sight, the absence of departure from mutation/drift equilibrium under the Wright-Fisher model (Figure 2B) would suggest the former hypothesis to be more supported and that the moderately low diversity of *O. edulis* cannot be simply attributed to a demographic effect. However, one might suspect that the assumptions of the Wright-Fisher model do not apply to highly fecund marine organisms (Eldon and Wakeley 2006, 2009; Sargsyan and Wakeley 2008). Non-Wright-Fisher models that consider multifurcating genealogical processes with or without simultaneous multiple mergers (Eldon and Wakeley 2006, 2009; Sargsyan and Wakeley 2008) might be more relevant to the *Ostrea* system, for which the hypothesis of sweepstakes reproductive success has already received support (Hedgecock *et al.* 2007). Under sweepstakes reproductive processes, Tajima’s D is expected to be negative at steady state (Sargsyan and Wakeley 2008). The fact that we observed it to be close to zero might therefore suggest that the population indeed departs from equilibrium. Another possibility could be that genetic differentiation among the geographically distant sampling locations we used could have contributed to affect the allele frequency spectrum (Städler *et al.* 2009; Cutter *et al.* 2012). However, *O. edulis* is known to be only slightly substructured, with an isolation-by-distance pattern (Launey *et al.* 2001). Not only we did not find evidence for population subdivision in our data but, under the hypothesis that there was indeed subdivision, the average silent diversity would have been inflated, while we found it to be low. Finally, selection on codon usage and functional noncoding DNA could have contributed to reducing silent diversity. We observed a similar level of diversity with synonymous and noncoding mutations, although synonymous diversity is usually lower than noncoding diversity in species in which synonymous selection is active (Zeng and Charlesworth 2010). Furthermore, we did not detect any correlation between diversity indices and codon bias. These results suggest that selection for codon usage is not very effective in *O. edulis* and corroborates the hypothesis of a low *Ne* on the long term. To summarize, *O. edulis* is not extremely polymorphic at silent sites and this is probably a consequence of its effective population size being smaller than expected from census size and previous measures of allozyme variability. Alternative hypotheses cannot be definitively ruled out, however, but most of them are expected to equally affect silent and nonsynonymous mutations.

### A high load of non-neutral amino acid polymorphisms

**Figure 3:**
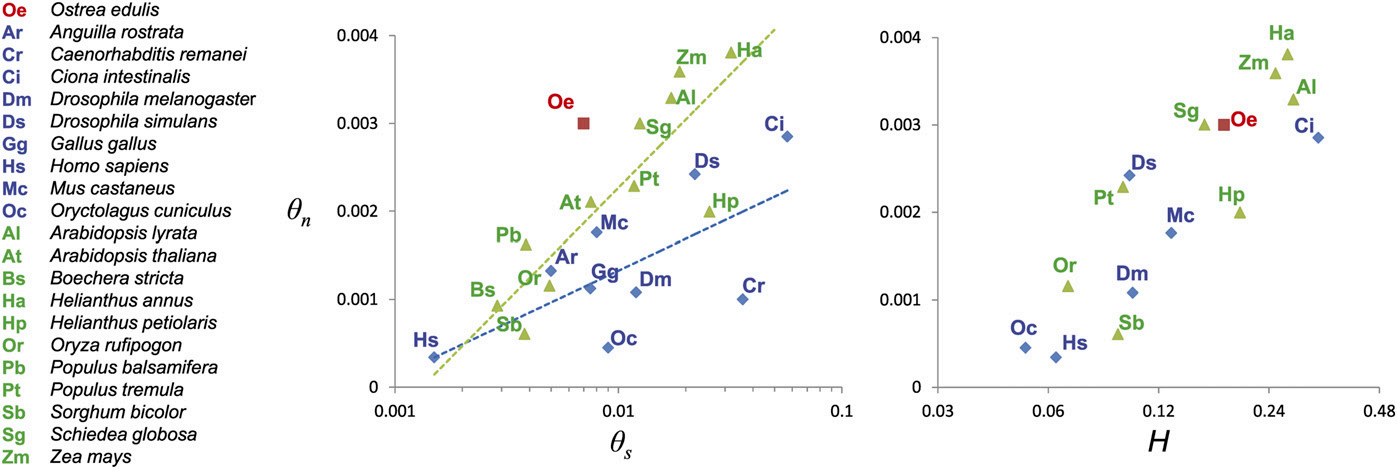
Nonsynonymous diversity (*θn*) in plant (green points) and animal species (red and blue points) as a function of synonymous diversity (*θs*, left panel) and as a function of allozyme heterozygosity (*H*, right panel). References used are listed in Table S4.

Although silent diversity was not extreme in *O. edulis*, a high level of nonsynonymous diversity was nonetheless observed (*π_n_* = 0.0024). High amino acid diversity is not restricted to a few extremely polymorphic proteins but appeared to be more a general feature of the whole collection of genes analyzed (Figure 1B). It is worth emphasizing that none of the examples of protein diversity previously reported to be extremely high (Moy *et al.* 2008; Wharam *et al.* 2008; Costa *et al.* 2009; Parisi *et al.* 2009) would appear as outliers if compared to the distribution we obtained in Figure 1B. Using the meta-analysis of Gossmann *et al.* (2010) in plants and some published results on nuclear DNA in animals (Fay *et al.* 2001; Bierne and Eyre-Walker 2004; Cutter 2008; Axelsson and Ellegren 2009; Halligan *et al.* 2010; Carneiro *et al.* 2012; Gagnaire *et al.* 2012; Tsagkogeorga *et al.* 2012), we computed *θ_s_* and *θ_n_* for a variety of species, shown in Figure 3. *θ_n_* seems greater than what could have been expected from silent diversity in *O. edulis* (Figure 3). On the other hand, this result supports high protein heterozygosity in marine bivalves. From the literature, we obtained the average allozyme heterozygosity (*H*) of all the possible species of Figure 3 (Schmidtke and Engel 1980; O’Brien *et al.* 1983; Nevo *et al.* 1984; Singh and Rhomberg 1987; Morden *et al.* 1989; Rajora and Dancik 1992; Saavedra *et al.* 1995; Weller *et al.* 1996; Cronn *et al.* 1997; Awasthi *et al.* 1998; Gao *et al.* 2000; Ansell *et al.* 2010) and plotted *θ_n_* against *H*. This time the flat oyster estimates fall right where they should be, in the group of species with the highest *θ_n_* and *H* values (Figure 3). Therefore, a discrepancy between silent and amino-acid diversities seems to exist, as already noted by Sauvage *et al.* (2007) in the cupped oyster *Crassostrea gigas*. As a consequence, the synonymous to nonsynonymous diversity ratio (*θ_n_*/*θ_s_*) observed in *O. edulis* was very high (*θ_n_*/*θ_s_* = 0.56), higher than any value we have seen in the literature to date. The explanation could be that the selective constraint is low (many amino acid mutations are neutral) and/or that there is a high segregating load in this species (many slightly selected mutations segregate in the populations). Partitioning nonsynonymous and silent SNPs according to their frequency, we estimated that 23% of amino acid changing mutations behave as neutral in *O. edulis*, which is similar to the estimates obtained in humans and the common fruit fly [20% and 24%, respectively (Fay *et al.* 2001; Shapiro *et al.* 2007)]. However, as much as 33% of nonsynonymous mutations are sufficiently weakly selected to segregate at low frequency in the polymorphism, which is much greater than usually reported. For instance, only 20% and 17% of amino acid changing mutations have been estimated to be mildly deleterious mutations in humans and flies, respectively. The high protein diversity of *O. edulis* is therefore partly due to a high load of segregating, weakly selected mutations. These mutations are often assumed to be slightly deleterious (Fay *et al.* 2001; Eyre-Walker *et al.* 2006), but any type of selection capable of generating an excess of low frequency amino acid variants can be invoked, such as balancing, weakly positive, or local selection, which may be more widespread than generally thought (Mitton 1997). Genetic incompatibilities (*i.e.*, negative epistatic interactions) are also often forgotten in population genomics (Fay 2011), even though they can accumulate in cryptic tension zones and contribute to the species-wide diversity (Bierne *et al.* 2011).

### How can we explain the discrepancy between protein and silent diversities?

Our results suggest that *O. edulis* might have greater protein diversity than expected from its effective size based on silent mutations. We can first suspect that the recent demography of *O. edulis* could be responsible for the high segregating load observed. When a population departs from demographic equilibrium, the allele frequency distribution of selected mutations is likely to be shifted more strongly than that of neutral mutations. Eyre-Walker *et al.* (2006) showed that this effect can be strong when the population is expanding while there is little effect in bottlenecked or admixed populations. The effect increases with the deviation, so that it is inevitably accompanied by a deviation of the synonymous frequency spectra (Eyre-Walker *et al.* 2006). We observed neither a departure from the Wright-Fisher model at equilibrium nor evidence of population subdivision in the *O. edulis* data. We conclude that demography should not explain the results obtained under the hypothesis of a Wright-Fisher functioning of *O. edulis* populations. If *O. edulis* really departs from demographic equilibrium, it would mean that it also departs from a Wright-Fisher model which is precisely the hypothesis we will propose hereafter.

The results obtained in *O. edulis* revitalize the hypothesis that the biological and ecological characteristics of marine invertebrates make their population genetics somewhat different from most other animal species, while making them more similar to these of highly fecund plants [see Williams’ “elm-oyster” model (Williams 1975; Launey and Hedgecock 2001)]. Many marine invertebrates share the characteristics of some plants, like trees: they are abundant, relatively long-lived and, maybe most importantly, highly fecund. Interestingly, Figure 3 suggests that the slope of the correlation between *θ_s_* and *θ_n_* tends to be slightly greater in plants and *O. edulis* than in animals. In oysters, adults produce millions of small eggs that develop into planktonic larvae that suffer high mortalities (Type III survivorship). Marine invertebrates undergo a large variance in reproductive success and generally have only fractions of the genetic diversity expected from their census size [see Hedgecock’s hypothesis of sweepstakes reproductive success (Hedgecock 1994; Hedgecock and Pudovkin 2011)]. In the same way that a large variance in reproductive success can impact the neutral coalescence process (Eldon and Wakeley 2006, 2009; Sargsyan and Wakeley 2008), it might also impact the behavior of selected mutations (Der *et al.* 2012). This does not mean that our estimate of the segregating load is flawed, as it was free from specific population genetics, but that the strong decoupling between *Ne* and *N* in non-Wright-Fisher populations with skewed offspring distribution could affect non-neutral mutations in an unpredicted manner. Theory predicts that the load of segregating deleterious mutations can deviate from the expectation based on a single Wright-Fisher population and become less dependent on the population size in subdivided populations (Glémin *et al.* 2003), and this deviation can become even stronger when the strength of selection vary spatially (Roze 2012) because of variation in the stressfulness of the environment or in source-sink metapopulation systems (Ronce and Kirkpatrick 2001).

The probability of fixation of a favorable mutation has recently been shown to be considerably increased when offspring distribution is skewed (Der *et al.* 2012). One can also speculate that deleterious mutations could remain polymorphic for longer periods as they could sometimes enter the population at a high frequency, “sweepstaking” in the progeny of a lucky winner and therefore taking more time to be purged than in a standard Wright-Fisher population. Investigating the effect of skewed offspring distribution on the evolution and diversity of deleterious mutations might be an interesting line for future research. In any case, one is left with the fact that high fecundity is a prerequisite for tolerating a high load of segregating selected mutations (Williams 1975). We should also emphasize here that the fecundity of *Ciona* sea squirts is two orders of magnitude lower than the fecundity of the flat oyster (Petersen and Svane 1995), which might explain the high *θ_s_* and low *θ_n_*/*θ_s_* ratio observed in these species. Quantifying the proportion of neutral and slightly selected amino acid mutations in other extremely fecund organisms will help to test the potential impact of fecundity and skewed offspring distribution on protein polymorphism and its relation with silent diversity.

### Comparing estimates of the genetic load obtained from molecular data and from segregation distortions in inbred progenies

The study of segregation distortion in inbred progenies has revealed a large number of strongly deleterious recessive mutations (~15 per genome on average) in the flat oyster *O. edulis* (Bierne *et al.* 1998) and cupped oyster *C. gigas* (Launey and Hedgecock 2001; Plough and Hedgecock 2011). In these experiments, a molecular marker typically maps ~40 cM of the genome. According to our estimate, ~170 non-neutral mutations are expected on this map length, although we have no idea of their true effect on viability. It is possible that only one mutation has a strong effect on fitness while the other mutations only have a negligible effect at the scale of the one generation used in these lab experiments. However, it is also possible that, assuming a single viability QTL at a given chromosomal position in mapping experiments is an unrealistic assumption that should deserve further examination in the future. The broad genomic distribution of distorted ratios (Lallias *et al.* 2007; Plough and Hedgecock 2011) might suggest a greater density of deleterious alleles than estimated, each with a lesser effect on fitness. One interpretation often neglected in QTL mapping is that of an abundance of mutations with small effects rather than a single mutation with a large effect, although this is highly plausible (Rockman 2011). Indeed, simulation studies of the infinitesimal model have shown that chance spatial clustering of infinitesimals caused by nonuniform recombination rates and gene densities can easily be confounded with a small number of large effect QTL (Noor *et al.* 2001). Our molecular estimate of a high genomic density of non-neutral amino-acid alleles in the *O. edulis* genome can be added to a long list of observations about the genetics of bivalve mollusks that started with abundant reports of heterozygosity-fitness correlations (Zouros 1987; Szulkin *et al.* 2010) and segregation distortion in pair crosses (Foltz 1986; Launey and Hedgecock 2001), which suggest bivalve genomes are heavily loaded by slightly selected polymorphisms. The architecture of this high genetic load will need to be further characterized and understood in order to correctly interpret the population genetics and evolution of these animals.

## Supplementary Material

Supporting Information
