# Supplementary material for: A High Load of Non-neutral Amino-Acid Polymorphisms Explains High Protein Diversity Despite Moderate Effective Population Size in a Marine Bivalve With Sweepstakes Reproduction
Source: G3 (Bethesda). 2013 Feb 1;3(2):333–41. doi: 10.1534/g3.112.005181 (PMC3564993; doi:10.1534/g3.112.005181)
Supplement: Supporting Information [file supp_3_2_333__index.html]

Supporting Information 

# A High Load of Non-neutral Amino-Acid Polymorphisms Explains High Protein Diversity Despite Moderate Effective Population Size in a Marine Bivalve With Sweepstakes Reproduction

## Supporting Information for Harrang *et al.*, 2013

**Files in this Data Supplement:**

- Supporting Information - Figures S1-S4 and Tables S1-S4 (PDF, 227 KB)
- Figure S1 - Functional classification of the EST sequences used for SNP identification (PDF, 96 KB)
- Figure S2 - Distribution of non-synonymous to silent diversity ratios of the 37 nuclear loci analyzed according to their putative biological function (PDF, 93 KB)
- Figure S3 - Distribution of (A) the Effective Number of codon (ENC) and (B) GC3 (GC content at third coding position) for the 37 nuclear loci analyzed in this study and for the 114 EST sequences (PDF, 79 KB)
- Figure S4 - Distribution of Fst values for silent (si) and non-synonymous (n) sites, (A) between all populations, (B) between Atlantic and Mediterranean populations (PDF, 79 KB)
- Table S1 - Primer sequences and size of the PCR products for the 40 loci of this study (PDF, 65 KB)
- Table S2 - Nucleotide diversity and Tajima's D statistic of the 40 loci analyzed in this study (PDF, 85 KB)
- Table S3 - Number of SNPs for the 37 nuclear loci analyzed in this study (PDF, 64 KB)
- Table S4 - References of published nuclear and allozyme data used in Figure 3 (PDF, 63 KB)
