# Supplementary material for: A High Load of Non-neutral Amino-Acid Polymorphisms Explains High Protein Diversity Despite Moderate Effective Population Size in a Marine Bivalve With Sweepstakes Reproduction
Source: G3 (Bethesda). 2013 Feb 1;3(2):333–41. doi: 10.1534/g3.112.005181 (PMC3564993; doi:10.1534/g3.112.005181)
Supplement: Supporting Information [file supp_3.2.333_005181SI.pdf]

G3 · Genes | Genomes | Genetics

A high load of non-neutral amino-acid polymorphisms explains high protein diversity despite moderate effective population size in a marine bivalve with sweepstakes reproduction

Estelle Harrang*, Sylvie Lapègue*, Benjamin Morga*, Nicolas Bierne[§,]**

* Ifremer, Laboratoire de génétique et pathologie, avenue de Mus de Loup 17390 La Tremblade, France
[§] Université Montpellier 2, Place Eugène Bataillon, 34095 Montpellier cedex 5, France
** ISEM - CNRS, UMR5554, Station Méditerranéenne de l'Environnement Littoral, 2 rue des Chantiers, 34200 Sète, France

[2]Corresponding author: Nicolas Bierne; Institut des Sciences de l'Evolution; Station Méditerranéenne de l'Environnement Littoral; 2, rue des Chantiers; 34200 Sète, France; Tel: 0033 467 46 33 75; Fax: 0033 467 46 33 99; E-Mail: n-bierne@univ-montp2.fr

DOI: 10.1534/g3.112.005181

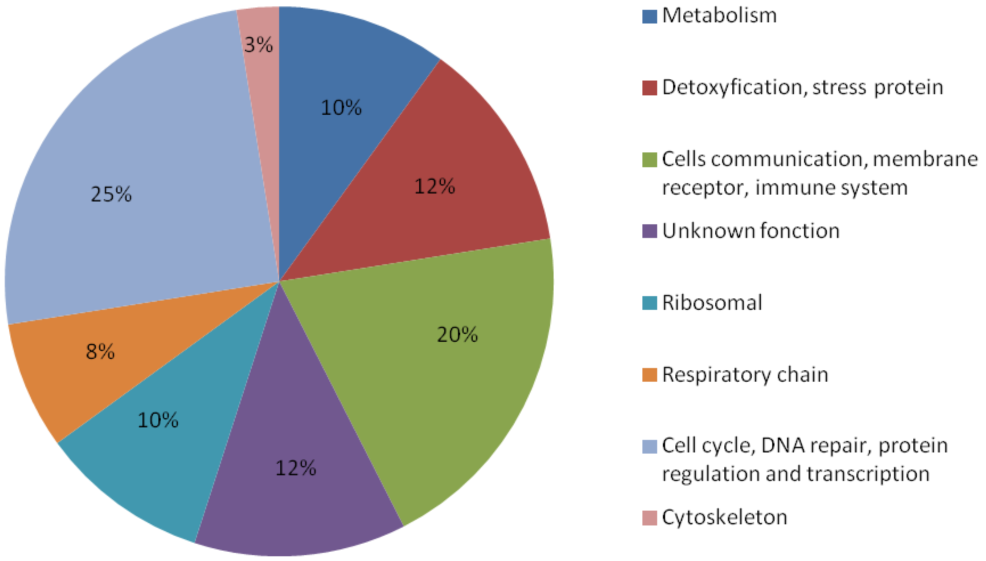

Figure S1 Functional classification of the EST sequences used for SNP identification. Genes were clustered into 8 categories according to their putative biological function.

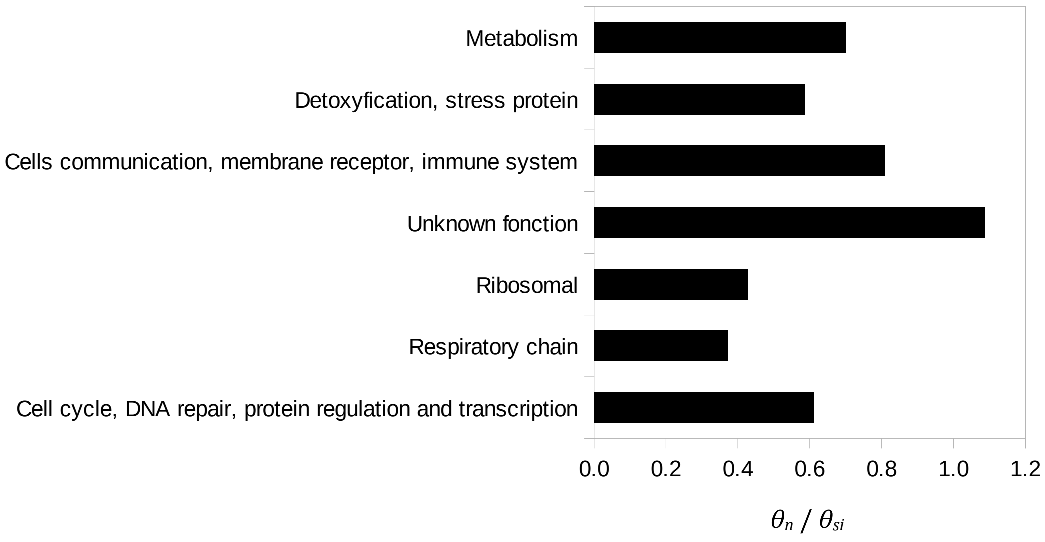

Figure S2 Distribution of non-synonymous to silent diversity ratios of the 37 nuclear loci analyzed according to their putative biological function.

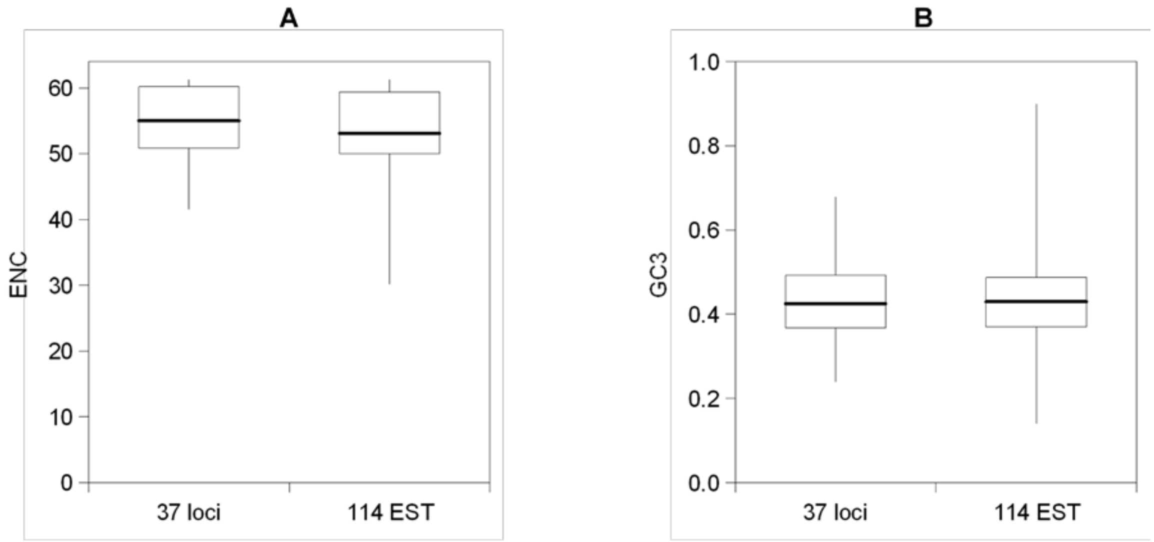

Figure S3 Distribution of (A) the Effective Number of codon (ENC) and (B) GC3 (GC content at third coding position) for the 37 nuclear loci analyzed in this study and for the 114 EST sequences.

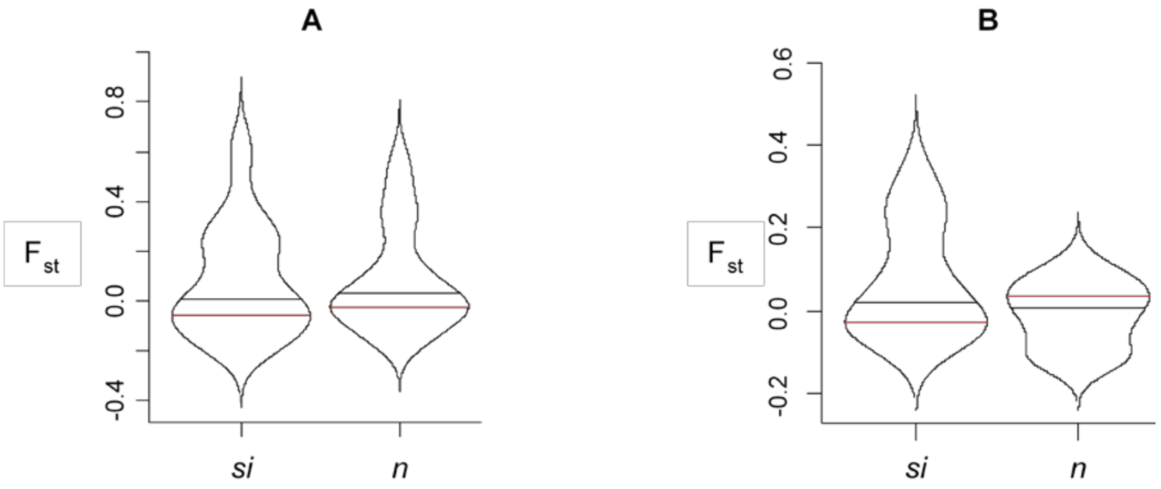

Figure S4 Distribution of Fst values for silent (si) and non-synonymous (n) sites, (A) between all populations, (B) between Atlantic and Mediterranean populations. Black lines are the means and red lines the modes of the distributions.

Table S1 Primer sequences and size of the PCR products for the 40 loci of this study.

GenBank Accession No.	Forward primer	Reverse primer	Product size (bp)	Size of the alignement used (bp)
JN680816	GTATCCCTGCCAAACAAAGC	TGGCTTTAGTGGCATTAGCA	390	390
JN680817	GGGGCCAGCTATAACATTCA	AACTGGAGGTCAGCAGCAAC	282	282
JN680818	GTCGTCAGGGGTCAGTTTGT	GCTGGCAAACTACGGGTTTA	1040	486
JN680819	GTCGGATGGTGAAAACAACC	CCTTTCCATCACCAGTGTCC	628	628
JN680820	TCAGTGCAGAGGTTCACAGG	CTTTGCACGGTTCCATCTCT	641	559
JN680821	ATGTACTCGCCCAGACCTGT	AACCCGACAGAGATGAGTGG	400	360
JN680822	ACCAGTTGTACGACCGGAAG	CCTTGCAGGGTGTTATGGTT	335	335
JN680823	AACCCTGACTGGTGGTATGC	CTGGGGAGGTCTCGCTTT	677	591
JN680824	GACGTAGGCTGCATCACTCA	GCCCTCCACTGAACATGAAT	925	865
JN680825	TCGAATTTTCTGCCGTCTTT	AGGCGGTGTGGATTGTCTAC	281	281
JN680826	GCCCAGGCTCTTTTACAGTG	TTACCACCAGCTCCCAGATT	297	297
JN680827	AGACCCCAATGTCACAGACC	CTCCTCATCCTCCAGCTCCT	607	607
JN680828	ACCAGAGGGAGGAAGAGAGC	CGTCAGTTTCTGGATGGTCA	343	343
JN680829	GGCCGAGGTACTTCTTCATC	TGGTGTTGTCGGATACATTGA	747	747
JN680830	CTGGCCTTACTGGAGACAAGA	ACCAAATTCGGGTACCCTCT	432	390
JN680831	CTGAACCCTTTGCATTTTCC	AGTTCGGACATGACTCTGCC	296	296
JN680832	GGGGGAAGGATGCAGAGT	GGACCGCAAATACTGGACA	256	256
JN680833	GGACCCACTTGAGCGTCTT	AGCTGCAACAGTTCCACGA	307	307
JN680834	ATGGAGCGAAGAGCAAGAAG	GATGTGGTTGTAATGGCACG	827	789
JN680835	CCTCTTTCCATGACCCATGT	CAGGTTGCAGAAACGGCTA	362	362
JN680836	TCCATGAGGACTCCACAAAT	GAACGGGTCAAAAAGAGAGG	200	200
JN680837	CCCAGTACAAGACCGGAAAA	TTCTCTCCTCCCAACTCGAA	860	850
JN680838	ACGCAATGAGGATGAAGAGG	GCCTTGTGGTACGGTGAACT	259	259
JN680839	GGACTTGAGTCAGTCGGAGGT	CTGGGGAGCAGAAGAGAGAC	292	292
JN680840	CCGAAGCTGCAGACGATT	GTTCCTCTCGGACTGTCCA	244	244
JN680841	AGTGATCAGCGGAAAGGCTA	AAGTCCTGGGACACAACGTC	657	657
JN680842	GCCTGGTGATTTATTTGCTTTC	GCAAAATGGACAGAAGAAGGAG	333	333
JN680843	GTGTGAGCAAATCGTGGAGA	GAGGACAATGCGGGAAAGTA	394	394
JN680844	GGCAAAACATCATCCCTCAG	TTCCCACAATCCACCACAC	380	380
JN680845	GTAACGTCAACGCCAACTT	TGAACCGAAAGTAGCCATCC	489	489
JN680846	ATTGGCATTGATCTCGGAAC	TTCATGTCGGACTGCACATT	245	245
JN680847	TTACACACCCGCCAATTTAAC	GCTGGAAAAGACTGTGAACCA	393	393
JN680848	CCAACTATCTTGGTGGGAACA	GCTTTTTGTTGGCTTTGGAC	228	228
JN680849	AACCTGTCTCACGACGGTCT	TACTCTACGCGAGGTTTCCG	301	301
JN680850	CCCTATGGAAAAGACCAGGAA	TAATTCCTTTGGGCACCAAG	857	728
JN680851	CCCTCAGAGCCTGTTCTGTT	GTGAAGAGGACCACGTCTGG	315	315
JN680852	CATTTCTATCTGGACCGGGA	CTCATCGCAAGTTCCCTTTC	713	679
JN680853	GTCGCTCACAGAAGCTGTACC	ATCATCTTGAACCACCCTGG	311	311
JN680854	CACCCAACAGAAGCTCATCC	CTGAGCACCTATGTGACTGTCC	592	432
JN680855	GGGGACGCCAGATTGTAA	GTCCACATCCTCGCCAAA	593	593

Table S2 Nucleotide diversity and Tajima's D statistic of the 40 loci analyzed in this study. n: non-synonymous sites; nc: non-coding sites; s: synonymous sites; si: silent sites. NS: non-significant; *: P < 0.05; **: P < 0.01. Linkage disequilibrium estimated by ZnS (KELLY 1997) is calculated for all SNPs and parsimony informative SNPs (PI SNPs).

GenBank	Kind of	Length regions (bp)					Nucleotide diversity					Waterson theta					Tajima D		ZnS	
accession no.	DNA	L	L_n	L_s	L_{nc}	L_{si}	π	π_n	π_s	π_{nc}	π_{si}	θ	θ_n	θ_s	θ_{nc}	θ_{si}	D	P-value	all SNPs	PI SNPs
**JN680818**	nDNA	486	0	0	486	486	0.0067			0.0067	0.0067	0.0078			0.0078	0.0078	-0.51	NS	0.213	0.239
**JN680819**	nDNA	628	188	52	388	440	0.0037	0.0066	0	0.0028	0.0025	0.0045	0.0067	0	0.0041	0.0036	-1.09	NS	0.094	0.107
**JN680820**	nDNA	559	143	37	379	416	0.0105	0.0023	0	0.0146	0.0133	0.0118	0.0031	0	0.0162	0.0148	-0.6	NS	0.619	0.750
**JN680821**	nDNA	360	155	40	162	202	0.0038	0.0040	0.0042	0.0037	0.0038	0.0068	0.0079	0.0062	0.0060	0.0061	-0.99	NS	0.108	0.138
**JN680822**	nDNA	335	257	76	0	76	0.0034	0	0.0148		0.0148	0.0028	0	0.0125		0.0125	0.58	NS	0.169	0.400
**JN680823**	nDNA	591	95	28	466	494	0.0019	0	0	0.0024	0.0023	0.0042	0	0	0.0053	0.0050	-1.83	*	0.124	0.022
**JN680824**	nDNA	865	124	41	700	741	0.0160	0.0058	0.0292	0.0171	0.0178	0.0130	0.0054	0.0242	0.0137	0.0143	1.09	NS	0.426	0.825
**JN680825**	nDNA	281	215	64	0	64	0.0007	0.0006	0.0007		0.0007	0.0017	0.0011	0.0036		0.0036	-1.12	NS	0.002	
**JN680827**	nDNA	607	169	50	386	436	0.0019	0	0	0.0030	0.0027	0.0014	0	0	0.0022	0.0019	1.04	NS	0.314	0.314
**JN680828**	nDNA	343	274	68	0	68	0	0	0		0	0	0	0		0				
**JN680829**	nDNA	747	168	45	531	576	0.0009	0.0020	0	0.0006	0.0006	0.0012	0.0026	0	0.0008	0.0008	-0.93	NS	0.040	
**JN680830**	nDNA	390	138	42	208	250	0.0034	0.0015	0.0025	0.0049	0.0045	0.0104	0.0069	0.0057	0.0137	0.0124	-2.01	*	0.211	0.476
**JN680831**	nDNA	296	223	71	0	71	0.0011	0.0012	0.0008		0.0008	0.0024	0.0022	0.0034		0.0034	-1.13	NS	0.078	
**JN680832**	nDNA	256	116	28	112	140	0.0017	0.0032	0	0.0004	0.0003	0.0055	0.0101	0	0.0021	0.0017	-1.12	NS	0.100	0.335
**JN680833**	nDNA	307	175	47	83	130	0.0023	0.0009	0.0097	0.0012	0.0043	0.0046	0.0040	0.0051	0.0057	0.0054	-0.48	NS	0.016	
**JN680834**	nDNA	789	147	39	603	642	0.0039	0.0023	0	0.0045	0.0043	0.0042	0.0045	0	0.0044	0.0041	0.12	NS	0.214	0.257
**JN680835**	nDNA	362	178	53	128	181	0.0022	0.0028	0	0.0022	0.0016	0.0035	0.0035	0	0.0049	0.0035	-1.48	NS	0.101	
**JN680836**	nDNA	200	154	44	0	44	0.0006	0.0007	0		0	0.0015	0.0019	0		0				
**JN680837**	nDNA	850	107	31	712	743	0.0043	0	0	0.0051	0.0049	0.0054	0	0	0.0065	0.0062	-0.96	NS	0.166	0.251
**JN680838**	nDNA	259	139	44	76	120	0.0047	0.0068	0	0.0038	0.0024	0.0073	0.0091	0	0.0083	0.0052	-1.48	NS	0.455	1
**JN680839**	nDNA	292	156	42	92	134	0.0009	0	0	0.0029	0.0020	0.0019	0	0	0.0060	0.0041	-1.17	NS	0.005	
**JN680840**	nDNA	244	192	48	0	48	0.0017	0.0022	0		0	0.0042	0.0054	0		0			0.003	
**JN680841**	nDNA	657	495	159	0	159	0.0020	0.0016	0.0030		0.0030	0.0032	0.0024	0.0057		0.0057	-1.35	NS	0.132	0.083
**JN680842**	nDNA	333	266	67	0	67	0.0019	0.0018	0.0021		0.0021	0.0046	0.0048	0.0039		0.0039	-0.74	NS	0.055	0.009
**JN680843**	nDNA	394	220	62	111	173	0.0020	0.0009	0.0044	0.0030	0.0035	0.0029	0.0026	0.0046	0.0025	0.0033	0.17	NS	0.015	0.044
**JN680844**	nDNA	380	124	35	219	254	0.0098	0	0.0187	0.0140	0.0147	0.0079	0	0.0173	0.0110	0.0119	0.88	NS	0.428	0.486
**JN680845**	nDNA	489	125	34	328	362	0.0025	0	0	0.0038	0.0034	0.0022	0	0	0.0032	0.0029	0.62	NS	0.080	0.078
**JN680846**	nDNA	245	188	55	0	55	0.0240	0.0079	0.0805		0.0805	0.0154	0.0062	0.0477		0.0477	2.43	*	0.470	0.470
**JN680847**	nDNA	393	161	43	188	231	0.0020	0.0007	0	0.0036	0.0029	0.0031	0.0030	0	0.0038	0.0031	-0.15	NS	0.016	0.066
**JN680848**	nDNA	228	174	54	0	54	0.0003	0	0.0012		0.0012	0.0011	0	0.0047		0.0047	-1.15	NS		
**JN680849**	nDNA	301	197	58	45	103	0.0016	0.0015	0.0032	0	0.0018	0.0028	0.0029	0.0048	0	0.0027	-0.59	NS	0.649	1
**JN680850**	nDNA	728	294	99	335	434	0.0032	0.0035	0.0058	0.0022	0.0031	0.0037	0.0039	0.0039	0.0035	0.0036	-0.63	NS	0.222	0.600
**JN680851**	nDNA	315	237	78	0	78	0.0141	0.0141	0.0142		0.0142	0.0090	0.0090	0.0090		0.0090	1.84	NS	1	1
**JN680852**	nDNA	679	255	75	348	423	0.0031	0.0023	0.0031	0.0036	0.0036	0.0044	0.0035	0.0040	0.0052	0.0050	-1.02	NS	0.258	0.349
**JN680853**	nDNA	311	123	36	149	185	0.0018	0.0007	0	0.0032	0.0026	0.0026	0.0022	0	0.0036	0.0029	-0.25	NS	0.049	
**JN680854**	nDNA	432	86	25	321	346	0.0064	0.0011	0.0195	0.0068	0.0077	0.0070	0.0032	0.0111	0.0077	0.0079	-0.19	NS	0.111	0.197
**JN680855**	nDNA	593	133	38	422	460	0.0137	0.0128	0	0.0152	0.0140	0.0102	0.0091	0	0.0114	0.0105	1.32	NS	0.339	0.494
**JN680816**	mtDNA	390	296	91		91	0.0050	0.0005	0.0196			0.0120	0.0009	0.0482			-2.08	*	0.342	0.187
**JN680817**	mtDNA	282	216	66		66	0.0046	0.0000	0.0198			0.0085	0	0.0365			-1.5	NS	0.378	0.057
**JN680826**	mtDNA	297	175	56	64	120	0.0053	0.0009	0.0216	0.0036	0.0205	0.0098	0.0026	0.0361	0.0070	0.0205	-1.22	NS	0.285	0.285
Total		17494	7278	2121	8042	10163														
Mean							0.0045	0.0024	0.0071	0.0050	0.0071	0.0054	0.0034	0.0076	0.0062	0.0069	-0.38		0.216	0.386

Table S3 Number of SNPs for the 37 nuclear loci analyzed in this study. n: non-synonymous SNPs; s: synonymous SNPs; nc: non-coding SNPs.

GenBank Accession no.	P	P_n	P_s	P_{nc}
JN680818	13			13
JN680819	9	4	0	5
JN680820	15	1	0	14
JN680821	10	5	1	4
JN680822	3	0	3	
JN680823	9	0	0	9
JN680824	34	2	3	29
JN680825	2	1	1	
JN680827	3	0	0	3
JN680828	0	0	0	
JN680829	2	1	0	1
JN680830	17	4	1	12
JN680831	3	2	1	
JN680832	6	5	0	1
JN680833	6	3	1	2
JN680834	10	2	0	8
JN680835	4	2	0	2
JN680836	1	1	0	
JN680837	13	0	0	13
JN680838	6	4	0	2
JN680839	2	0	0	2
JN680840	4	4	0	
JN680841	7	4	3	
JN680842	6	5	1	
JN680843	4	2	1	1
JN680844	10	0	2	8
JN680845	3	0	0	3
JN680846	13	4	9	
JN680847	5	2	0	3
JN680848	1	0	1	
JN680849	3	2	1	0
JN680850	7	3	1	3
JN680851	8	6	2	
JN680852	10	3	1	6
JN680853	3	1	0	2
JN680854	11	1	1	9
JN680855	20	4	0	16
Total	283	78	34	171

Table S4 References of published nuclear and allozyme data used in Figure 3.

Species	Nuclear data	Allozyme data
Anguilla rostrata	Gagnaire *et al*. (2012)	
Caenorhabditis remanei	Cutter (2008)	
Ciona intestinalis	Tsagkogeorga *et al.* (2012)	Schmidtke and Engel (1980)
Drosophila melanogaster	Bierne and Eyre-Walker (2004)	Singh and Rhomberg (1987)
Drosophila simulans	Bierne and Eyre-Walker (2004)	Singh and Rhomberg (1987)
Gallus gallus	Axelsson and Ellegren (2009)	
Homo sapiens	Fay *et al.* (2001)	O'Brien *et al.* (1983)
Mus castaneus	Halligan *et al.* (2010)	Awasthi *et al.* (1998)
Oryctolagus cuniculus	Carneiro *et al.* (2012)	Nevo *et al.* (1984)
Arabidopsis lyrata	Gossmann *et al.* (2010)	Ansell *et al.* (2010)
Arabidopsis thaliana	Gossmann *et al.* (2010)	
Boechera stricta	Gossmann *et al.* (2010)	
Helianthus annus	Gossmann *et al.* (2010)	Cronn *et al.* (1997)
Helianthus petiolaris	Gossmann *et al.* (2010)	Cronn *et al.* (1997)
Oryza rufipogon	Gossmann *et al.* (2010)	Gao and Hong (2000)
Populus balsamifera	Gossmann *et al.* (2010)	
Populus tremula	Gossmann *et al.* (2010)	Rajora and Dancik (1992)
Schiedea globosa	Gossmann *et al.* (2010)	Weller *et al.* (1996)
Sorghum bicolor	Gossmann *et al.* (2010)	Morden *et al.* (1989)
Zea mays	Gossmann *et al.* (2010)	Cronn *et al.* (1997)
Ostrea edulis	This study	Saavedra *et al.* (1995)
