# Supplementary material for: A High Load of Non-neutral Amino-Acid Polymorphisms Explains High Protein Diversity Despite Moderate Effective Population Size in a Marine Bivalve With Sweepstakes Reproduction
Source: G3 (Bethesda). 2013 Feb 1;3(2):333–41. doi: 10.1534/g3.112.005181 (PMC3564993; doi:10.1534/g3.112.005181)
Supplement: Supporting Information [file supp_3.2.333_FigureS3.pdf]

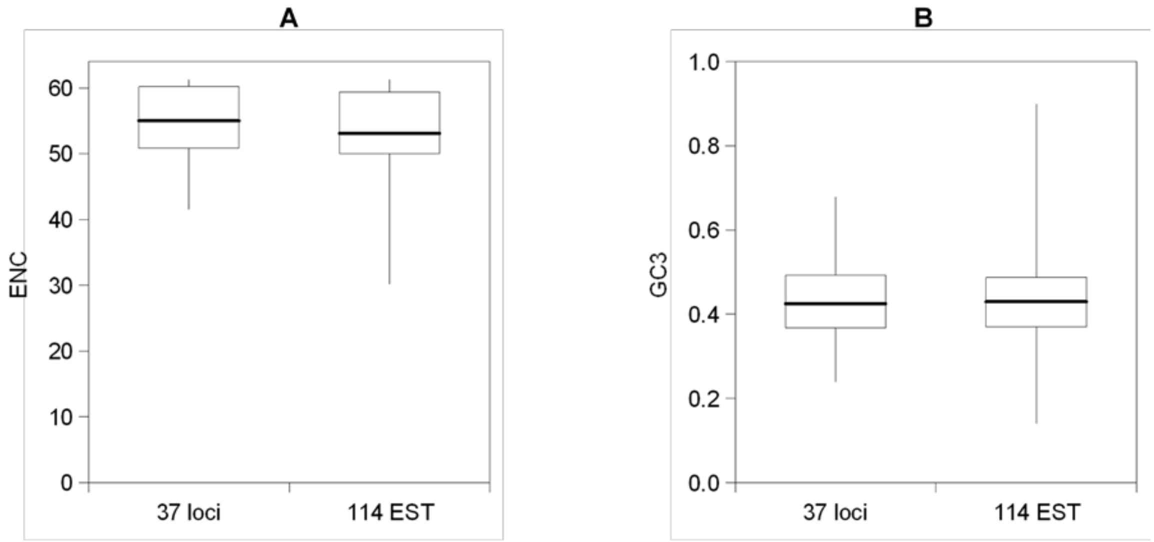

Figure S3 Distribution of (A) the Effective Number of codon (ENC) and (B) GC3 (GC content at third coding position) for the 37 nuclear loci analyzed in this study and for the 114 EST sequences.
