# Supplementary material for: A High Load of Non-neutral Amino-Acid Polymorphisms Explains High Protein Diversity Despite Moderate Effective Population Size in a Marine Bivalve With Sweepstakes Reproduction
Source: G3 (Bethesda). 2013 Feb 1;3(2):333–41. doi: 10.1534/g3.112.005181 (PMC3564993; doi:10.1534/g3.112.005181)
Supplement: Supporting Information [file supp_3.2.333_FigureS4.pdf]

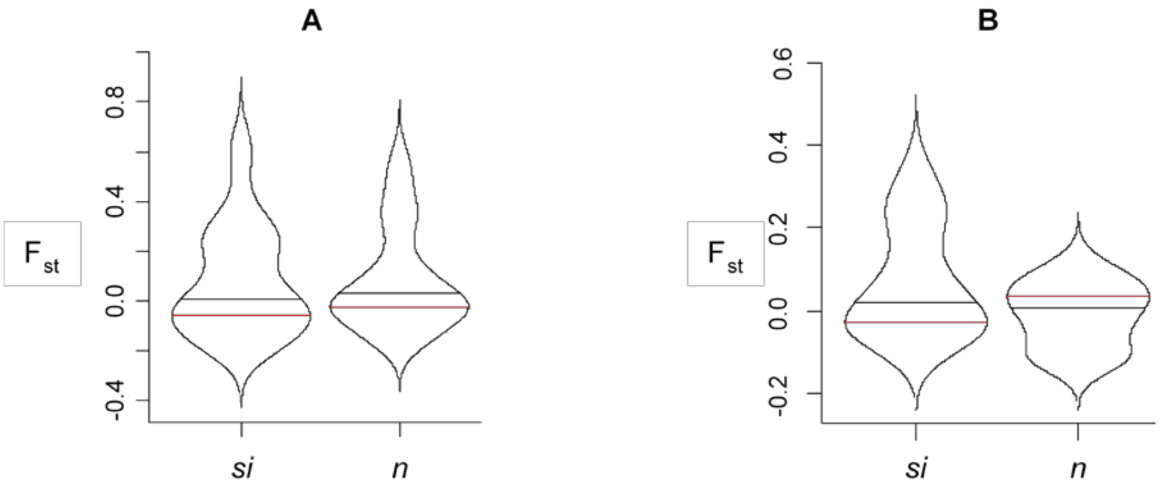

Figure S4 Distribution of Fst values for silent (si) and non-synonymous (n) sites, (A) between all populations, (B) between Atlantic and Mediterranean populations. Black lines are the means and red lines the modes of the distributions.
