# Supplementary material for: A High Load of Non-neutral Amino-Acid Polymorphisms Explains High Protein Diversity Despite Moderate Effective Population Size in a Marine Bivalve With Sweepstakes Reproduction
Source: G3 (Bethesda). 2013 Feb 1;3(2):333–41. doi: 10.1534/g3.112.005181 (PMC3564993; doi:10.1534/g3.112.005181)
Supplement: Supporting Information [file supp_3.2.333_TableS1.pdf]

Table S1 Primer sequences and size of the PCR products for the 40 loci of this study.

GenBank Accession No.	Forward primer	Reverse primer	Product size (bp)	Size of the alignement used (bp)
JN680816	GTATCCCTGCCAAACAAAGC	TGGCTTTAGTGGCATTAGCA	390	390
JN680817	GGGGCCAGCTATAACATTCA	AACTGGAGGTCAGCAGCAAC	282	282
JN680818	GTCGTCAGGGGTCAGTTTGT	GCTGGCAAACTACGGGTTTA	1040	486
JN680819	GTCGGATGGTGAAAACAACC	CCTTTCCATCACCAGTGTCC	628	628
JN680820	TCAGTGCAGAGGTTCACAGG	CTTTGCACGGTTCCATCTCT	641	559
JN680821	ATGTACTCGCCCAGACCTGT	AACCCGACAGAGATGAGTGG	400	360
JN680822	ACCAGTTGTACGACCGGAAG	CCTTGCAGGGTGTTATGGTT	335	335
JN680823	AACCCTGACTGGTGGTATGC	CTGGGGAGGTCTCGCTTT	677	591
JN680824	GACGTAGGCTGCATCACTCA	GCCCTCCACTGAACATGAAT	925	865
JN680825	TCGAATTTTCTGCCGTCTTT	AGGCGGTGTGGATTGTCTAC	281	281
JN680826	GCCCAGGCTCTTTTACAGTG	TTACCACCAGCTCCCAGATT	297	297
JN680827	AGACCCCAATGTCACAGACC	CTCCTCATCCTCCAGCTCCT	607	607
JN680828	ACCAGAGGGAGGAAGAGAGC	CGTCAGTTTCTGGATGGTCA	343	343
JN680829	GGCCGAGGTACTTCTTCATC	TGGTGTTGTCGGATACATTGA	747	747
JN680830	CTGGCCTTACTGGAGACAAGA	ACCAAATTCGGGTACCCTCT	432	390
JN680831	CTGAACCCTTTGCATTTTCC	AGTTCGGACATGACTCTGCC	296	296
JN680832	GGGGGAAGGATGCAGAGT	GGACCGCAAATACTGGACA	256	256
JN680833	GGACCCACTTGAGCGTCTT	AGCTGCAACAGTTCCACGA	307	307
JN680834	ATGGAGCGAAGAGCAAGAAG	GATGTGGTTGTAATGGCACG	827	789
JN680835	CCTCTTTCCATGACCCATGT	CAGGTTGCAGAAACGGCTA	362	362
JN680836	TCCATGAGGACTCCACAAAT	GAACGGGTCAAAAAGAGAGG	200	200
JN680837	CCCAGTACAAGACCGGAAAA	TTCTCTCCTCCCAACTCGAA	860	850
JN680838	ACGCAATGAGGATGAAGAGG	GCCTTGTGGTACGGTGAACT	259	259
JN680839	GGACTTGAGTCAGTCGGAGGT	CTGGGGAGCAGAAGAGAGAC	292	292
JN680840	CCGAAGCTGCAGACGATT	GTTCCTCTCGGACTGTCCA	244	244
JN680841	AGTGATCAGCGGAAAGGCTA	AAGTCCTGGGACACAACGTC	657	657
JN680842	GCCTGGTGATTTATTTGCTTTC	GCAAAATGGACAGAAGAAGGAG	333	333
JN680843	GTGTGAGCAAATCGTGGAGA	GAGGACAATGCGGGAAAGTA	394	394
JN680844	GGCAAAACATCATCCCTCAG	TTCCCACAATCCACCACAC	380	380
JN680845	GTAACGTCAACGCCAACTT	TGAACCGAAAGTAGCCATCC	489	489
JN680846	ATTGGCATTGATCTCGGAAC	TTCATGTCGGACTGCACATT	245	245
JN680847	TTACACACCCGCCAATTTAAC	GCTGGAAAAGACTGTGAACCA	393	393
JN680848	CCAACTATCTTGGTGGGAACA	GCTTTTTGTTGGCTTTGGAC	228	228
JN680849	AACCTGTCTCACGACGGTCT	TACTCTACGCGAGGTTTCCG	301	301
JN680850	CCCTATGGAAAAGACCAGGAA	TAATTCCTTTGGGCACCAAG	857	728
JN680851	CCCTCAGAGCCTGTTCTGTT	GTGAAGAGGACCACGTCTGG	315	315
JN680852	CATTTCTATCTGGACCGGGA	CTCATCGCAAGTTCCCTTTC	713	679
JN680853	GTCGCTCACAGAAGCTGTACC	ATCATCTTGAACCACCCTGG	311	311
JN680854	CACCCAACAGAAGCTCATCC	CTGAGCACCTATGTGACTGTCC	592	432
JN680855	GGGGACGCCAGATTGTAA	GTCCACATCCTCGCCAAA	593	593
