# Supplementary material for: A High Load of Non-neutral Amino-Acid Polymorphisms Explains High Protein Diversity Despite Moderate Effective Population Size in a Marine Bivalve With Sweepstakes Reproduction
Source: G3 (Bethesda). 2013 Feb 1;3(2):333–41. doi: 10.1534/g3.112.005181 (PMC3564993; doi:10.1534/g3.112.005181)
Supplement: Supporting Information [file supp_3.2.333_TableS2.pdf]

Table S2 Nucleotide diversity and Tajima's D statistic of the 40 loci analyzed in this study. n: non-synonymous sites; nc: non-coding sites; s: synonymous sites; si: silent sites. NS: non-significant; *: P < 0.05; **: P < 0.01. Linkage disequilibrium estimated by ZnS (KELLY 1997) is calculated for all SNPs and parsimony informative SNPs (PI SNPs).

GenBank	Kind of	Length regions (bp)					Nucleotide diversity					Waterson theta					Tajima D		ZnS	
accession no.	DNA	L	L_n	L_s	L_{nc}	L_{si}	π	π_n	π_s	π_{nc}	π_{si}	θ	θ_n	θ_s	θ_{nc}	θ_{si}	D	P-value	all SNPs	PI SNPs
**JN680818**	nDNA	486	0	0	486	486	0.0067			0.0067	0.0067	0.0078			0.0078	0.0078	-0.51	NS	0.213	0.239
**JN680819**	nDNA	628	188	52	388	440	0.0037	0.0066	0	0.0028	0.0025	0.0045	0.0067	0	0.0041	0.0036	-1.09	NS	0.094	0.107
**JN680820**	nDNA	559	143	37	379	416	0.0105	0.0023	0	0.0146	0.0133	0.0118	0.0031	0	0.0162	0.0148	-0.6	NS	0.619	0.750
**JN680821**	nDNA	360	155	40	162	202	0.0038	0.0040	0.0042	0.0037	0.0038	0.0068	0.0079	0.0062	0.0060	0.0061	-0.99	NS	0.108	0.138
**JN680822**	nDNA	335	257	76	0	76	0.0034	0	0.0148		0.0148	0.0028	0	0.0125		0.0125	0.58	NS	0.169	0.400
**JN680823**	nDNA	591	95	28	466	494	0.0019	0	0	0.0024	0.0023	0.0042	0	0	0.0053	0.0050	-1.83	*	0.124	0.022
**JN680824**	nDNA	865	124	41	700	741	0.0160	0.0058	0.0292	0.0171	0.0178	0.0130	0.0054	0.0242	0.0137	0.0143	1.09	NS	0.426	0.825
**JN680825**	nDNA	281	215	64	0	64	0.0007	0.0006	0.0007		0.0007	0.0017	0.0011	0.0036		0.0036	-1.12	NS	0.002	
**JN680827**	nDNA	607	169	50	386	436	0.0019	0	0	0.0030	0.0027	0.0014	0	0	0.0022	0.0019	1.04	NS	0.314	0.314
**JN680828**	nDNA	343	274	68	0	68	0	0	0		0	0	0	0		0				
**JN680829**	nDNA	747	168	45	531	576	0.0009	0.0020	0	0.0006	0.0006	0.0012	0.0026	0	0.0008	0.0008	-0.93	NS	0.040	
**JN680830**	nDNA	390	138	42	208	250	0.0034	0.0015	0.0025	0.0049	0.0045	0.0104	0.0069	0.0057	0.0137	0.0124	-2.01	*	0.211	0.476
**JN680831**	nDNA	296	223	71	0	71	0.0011	0.0012	0.0008		0.0008	0.0024	0.0022	0.0034		0.0034	-1.13	NS	0.078	
**JN680832**	nDNA	256	116	28	112	140	0.0017	0.0032	0	0.0004	0.0003	0.0055	0.0101	0	0.0021	0.0017	-1.12	NS	0.100	0.335
**JN680833**	nDNA	307	175	47	83	130	0.0023	0.0009	0.0097	0.0012	0.0043	0.0046	0.0040	0.0051	0.0057	0.0054	-0.48	NS	0.016	
**JN680834**	nDNA	789	147	39	603	642	0.0039	0.0023	0	0.0045	0.0043	0.0042	0.0045	0	0.0044	0.0041	0.12	NS	0.214	0.257
**JN680835**	nDNA	362	178	53	128	181	0.0022	0.0028	0	0.0022	0.0016	0.0035	0.0035	0	0.0049	0.0035	-1.48	NS	0.101	
**JN680836**	nDNA	200	154	44	0	44	0.0006	0.0007	0		0	0.0015	0.0019	0		0				
**JN680837**	nDNA	850	107	31	712	743	0.0043	0	0	0.0051	0.0049	0.0054	0	0	0.0065	0.0062	-0.96	NS	0.166	0.251
**JN680838**	nDNA	259	139	44	76	120	0.0047	0.0068	0	0.0038	0.0024	0.0073	0.0091	0	0.0083	0.0052	-1.48	NS	0.455	1
**JN680839**	nDNA	292	156	42	92	134	0.0009	0	0	0.0029	0.0020	0.0019	0	0	0.0060	0.0041	-1.17	NS	0.005	
**JN680840**	nDNA	244	192	48	0	48	0.0017	0.0022	0		0	0.0042	0.0054	0		0			0.003	
**JN680841**	nDNA	657	495	159	0	159	0.0020	0.0016	0.0030		0.0030	0.0032	0.0024	0.0057		0.0057	-1.35	NS	0.132	0.083
**JN680842**	nDNA	333	266	67	0	67	0.0019	0.0018	0.0021		0.0021	0.0046	0.0048	0.0039		0.0039	-0.74	NS	0.055	0.009
**JN680843**	nDNA	394	220	62	111	173	0.0020	0.0009	0.0044	0.0030	0.0035	0.0029	0.0026	0.0046	0.0025	0.0033	0.17	NS	0.015	0.044
**JN680844**	nDNA	380	124	35	219	254	0.0098	0	0.0187	0.0140	0.0147	0.0079	0	0.0173	0.0110	0.0119	0.88	NS	0.428	0.486
**JN680845**	nDNA	489	125	34	328	362	0.0025	0	0	0.0038	0.0034	0.0022	0	0	0.0032	0.0029	0.62	NS	0.080	0.078
**JN680846**	nDNA	245	188	55	0	55	0.0240	0.0079	0.0805		0.0805	0.0154	0.0062	0.0477		0.0477	2.43	*	0.470	0.470
**JN680847**	nDNA	393	161	43	188	231	0.0020	0.0007	0	0.0036	0.0029	0.0031	0.0030	0	0.0038	0.0031	-0.15	NS	0.016	0.066
**JN680848**	nDNA	228	174	54	0	54	0.0003	0	0.0012		0.0012	0.0011	0	0.0047		0.0047	-1.15	NS		
**JN680849**	nDNA	301	197	58	45	103	0.0016	0.0015	0.0032	0	0.0018	0.0028	0.0029	0.0048	0	0.0027	-0.59	NS	0.649	1
**JN680850**	nDNA	728	294	99	335	434	0.0032	0.0035	0.0058	0.0022	0.0031	0.0037	0.0039	0.0039	0.0035	0.0036	-0.63	NS	0.222	0.600
**JN680851**	nDNA	315	237	78	0	78	0.0141	0.0141	0.0142		0.0142	0.0090	0.0090	0.0090		0.0090	1.84	NS	1	1
**JN680852**	nDNA	679	255	75	348	423	0.0031	0.0023	0.0031	0.0036	0.0036	0.0044	0.0035	0.0040	0.0052	0.0050	-1.02	NS	0.258	0.349
**JN680853**	nDNA	311	123	36	149	185	0.0018	0.0007	0	0.0032	0.0026	0.0026	0.0022	0	0.0036	0.0029	-0.25	NS	0.049	
**JN680854**	nDNA	432	86	25	321	346	0.0064	0.0011	0.0195	0.0068	0.0077	0.0070	0.0032	0.0111	0.0077	0.0079	-0.19	NS	0.111	0.197
**JN680855**	nDNA	593	133	38	422	460	0.0137	0.0128	0	0.0152	0.0140	0.0102	0.0091	0	0.0114	0.0105	1.32	NS	0.339	0.494
**JN680816**	mtDNA	390	296	91		91	0.0050	0.0005	0.0196			0.0120	0.0009	0.0482			-2.08	*	0.342	0.187
**JN680817**	mtDNA	282	216	66		66	0.0046	0.0000	0.0198			0.0085	0	0.0365			-1.5	NS	0.378	0.057
**JN680826**	mtDNA	297	175	56	64	120	0.0053	0.0009	0.0216	0.0036	0.0205	0.0098	0.0026	0.0361	0.0070	0.0205	-1.22	NS	0.285	0.285
Total		17494	7278	2121	8042	10163														
Mean							0.0045	0.0024	0.0071	0.0050	0.0071	0.0054	0.0034	0.0076	0.0062	0.0069	-0.38		0.216	0.386
