# Supplementary material for: A High Load of Non-neutral Amino-Acid Polymorphisms Explains High Protein Diversity Despite Moderate Effective Population Size in a Marine Bivalve With Sweepstakes Reproduction
Source: G3 (Bethesda). 2013 Feb 1;3(2):333–41. doi: 10.1534/g3.112.005181 (PMC3564993; doi:10.1534/g3.112.005181)
Supplement: Supporting Information [file supp_3.2.333_TableS3.pdf]

Table S3 Number of SNPs for the 37 nuclear loci analyzed in this study. n: non-synonymous SNPs; s: synonymous SNPs; nc: non-coding SNPs.

GenBank Accession no.	P	P_n	P_s	P_{nc}
JN680818	13			13
JN680819	9	4	0	5
JN680820	15	1	0	14
JN680821	10	5	1	4
JN680822	3	0	3	
JN680823	9	0	0	9
JN680824	34	2	3	29
JN680825	2	1	1	
JN680827	3	0	0	3
JN680828	0	0	0	
JN680829	2	1	0	1
JN680830	17	4	1	12
JN680831	3	2	1	
JN680832	6	5	0	1
JN680833	6	3	1	2
JN680834	10	2	0	8
JN680835	4	2	0	2
JN680836	1	1	0	
JN680837	13	0	0	13
JN680838	6	4	0	2
JN680839	2	0	0	2
JN680840	4	4	0	
JN680841	7	4	3	
JN680842	6	5	1	
JN680843	4	2	1	1
JN680844	10	0	2	8
JN680845	3	0	0	3
JN680846	13	4	9	
JN680847	5	2	0	3
JN680848	1	0	1	
JN680849	3	2	1	0
JN680850	7	3	1	3
JN680851	8	6	2	
JN680852	10	3	1	6
JN680853	3	1	0	2
JN680854	11	1	1	9
JN680855	20	4	0	16
Total	283	78	34	171
