# Supplementary material for: A High Load of Non-neutral Amino-Acid Polymorphisms Explains High Protein Diversity Despite Moderate Effective Population Size in a Marine Bivalve With Sweepstakes Reproduction
Source: G3 (Bethesda). 2013 Feb 1;3(2):333–41. doi: 10.1534/g3.112.005181 (PMC3564993; doi:10.1534/g3.112.005181)
Supplement: Supporting Information [file supp_3.2.333_TableS4.pdf]

Table S4 References of published nuclear and allozyme data used in Figure 3.

Species	Nuclear data	Allozyme data
Anguilla rostrata	Gagnaire *et al*. (2012)	
Caenorhabditis remanei	Cutter (2008)	
Ciona intestinalis	Tsagkogeorga *et al.* (2012)	Schmidtke and Engel (1980)
Drosophila melanogaster	Bierne and Eyre-Walker (2004)	Singh and Rhomberg (1987)
Drosophila simulans	Bierne and Eyre-Walker (2004)	Singh and Rhomberg (1987)
Gallus gallus	Axelsson and Ellegren (2009)	
Homo sapiens	Fay *et al.* (2001)	O'Brien *et al.* (1983)
Mus castaneus	Halligan *et al.* (2010)	Awasthi *et al.* (1998)
Oryctolagus cuniculus	Carneiro *et al.* (2012)	Nevo *et al.* (1984)
Arabidopsis lyrata	Gossmann *et al.* (2010)	Ansell *et al.* (2010)
Arabidopsis thaliana	Gossmann *et al.* (2010)	
Boechera stricta	Gossmann *et al.* (2010)	
Helianthus annus	Gossmann *et al.* (2010)	Cronn *et al.* (1997)
Helianthus petiolaris	Gossmann *et al.* (2010)	Cronn *et al.* (1997)
Oryza rufipogon	Gossmann *et al.* (2010)	Gao and Hong (2000)
Populus balsamifera	Gossmann *et al.* (2010)	
Populus tremula	Gossmann *et al.* (2010)	Rajora and Dancik (1992)
Schiedea globosa	Gossmann *et al.* (2010)	Weller *et al.* (1996)
Sorghum bicolor	Gossmann *et al.* (2010)	Morden *et al.* (1989)
Zea mays	Gossmann *et al.* (2010)	Cronn *et al.* (1997)
Ostrea edulis	This study	Saavedra *et al.* (1995)
